# COVID‐19 vaccine hesitancy and attitudes in Qatar: A national cross‐sectional survey of a migrant‐majority population

**DOI:** 10.1111/irv.12847

**Published:** 2021-02-19

**Authors:** Majid Alabdulla, Shuja Mohd Reagu, Abdullatif Al‐Khal, Marwa Elzain, Roland M. Jones

**Affiliations:** ^1^ Hamad Medical Corporation Doha Qatar; ^2^ College of Medicine Qatar University Doha Qatar; ^3^ Centre for Addiction and Mental Health and University of Toronto Toronto ON Canada

**Keywords:** COVID‐19, COVID‐19 vaccine, migrant mental health, Qatar, social media, vaccine hesitancy

## Abstract

**Background:**

Vaccine hesitancy is a global threat undermining control of preventable infections. Emerging evidence suggests that hesitancy to COVID‐19 vaccination varies globally. Qatar has a unique population with around 90% of the population being economic migrants, and the degree and determinants of hesitancy are not known.

**Methods:**

This study was carried out to evaluate the degree of vaccine hesitancy and its socio‐demographic and attitudinal determinants across a representative sample. A national cross‐sectional study using validated hesitancy measurement tool was carried out from October 15, 2020, to November 15, 2020. A total of 7821 adults completed the survey. Relevant socio‐demographic data along with attitudes and beliefs around COVID‐19 vaccination were collected from the respondents.

**Results:**

20.2% of the respondents stated they would not take the vaccine and 19.8% reported being unsure about taking the prospective COVID‐19 vaccine. Citizens and females were more likely to be vaccine hesitators than immigrants and males, respectively. Concerns around the safety of COVID‐19 vaccine and its longer‐term side effects were the main concerns cited. Personal research around COVID‐19 and vaccine were by far the most preferred methods that would increase confidence in accepting the vaccine across all demographic groups.

**Conclusions:**

This study reports an overall vaccine hesitancy of 20% toward the COVID‐19 vaccine and the influence of social media on attitudes toward vaccination which is in keeping with emerging evidence. This finding comes at a time that is close to the start of mass immunization and reports from a migrant‐majority population highlighting important socio‐demographic determinants around vaccine hesitancy.

## 1 INTRODUCTION

COVID‐19 was declared a global pandemic by the WHO in March 2020.^1^ Toward the end of November 2020, the pandemic had spread to 215 countries and territories, infecting over 61 102 236 people, causing 1 433 132 deaths^2^ and had an unprecedented negative impact on economic activity, education, travel, food production, and social activities.^3^, ^4^, ^5^, ^6^ Despite a global effort to evaluate treatments for COVID‐19, no anti‐viral agents have yet been identified as effective treatment.^7^, ^8^ Development of an effective vaccine to provide immunization was therefore identified early on as the main exit strategy from this global pandemic.^9^ As a result, multiple vaccine development programs across the world have been working to develop an effective vaccine for COVID‐19, and as of the end of November 2020, at least 55 vaccines were undergoing clinical trials on humans with at least 3 seeking approval for public use.^10^

Vaccination requires inoculating a certain proportion of the at‐risk population in order to achieve immunity of the whole population.^11^ Despite being acknowledged as one of the most successful public health measures, many individuals choose not to be vaccinated citing concerns around safety and questioning the necessity of immunization, and there is evidence that vaccine refusal and hesitancy by individuals across the world have been increasing.^12^, ^13^ This hesitancy has been recognized as one of the top ten global health threats by the WHO.^14^

The WHO set up the Strategic Advisory Group of Experts (SAGE) to address the global challenge of vaccine hesitancy and refusal. Studies by SAGE and other research groups have identified a number of reasons that may contribute to vaccine hesitancy; and although these reasons may vary across different countries, vaccine hesitancy is recognized as a growing concern.^15^, ^16^

Reports are emerging from several countries across the world, exploring attitudes to the prospective COVID‐19 vaccine.^17^, ^18^, ^19^, ^20^, ^21^, ^22^ The results from these studies show high levels of vaccine hesitancy to COVID‐19 vaccination, ranging from 20% to 40% of the surveyed populations. Most of the published studies are based in developed economies with majority native/local populations.

Qatar is a hub of international travel and massive economic development and incidentally has had one of the highest COVID‐19 infection rates in the world.^23^, ^24^ Therefore, like other countries, vaccination will play a major role in managing the effects of this pandemic in this nation. Qatar has a unique population, in that the over 90% of the residents are economic immigrants from other countries.^25^ The concerns and priorities of this migrant‐majority population, who do not live with their wider families or elderly relatives and are keen to return back to economic activity, are likely to differ. We anticipated that the motivations for accepting vaccines will therefore be different in Qatar compared to the native majority populations studied to date. Additionally, there are no studies on COVID‐19 vaccine hesitancy from Qatar and the wider MENA region, with similar demographic characteristics, to our knowledge.

Therefore, it is important to explore attitudes toward vaccination and the degree and nature of potential vaccine refusal. We carried out a national cross‐sectional survey of adults in the State of Qatar to measure attitudes toward COVID‐19 vaccination using a validated vaccine hesitancy tool and to study socio‐demographic and personality characteristics associated with vaccine hesitancy. In this article, we present a subset of the findings of the wider study focusing on the degree of COVID‐19 vaccine hesitancy and its socio‐demographic correlates.

## 2 METHODOLOGY

### 2.1 Study design

We conducted a national cross‐sectional survey in Qatar between October 15 and November 15, 2020 using an online survey. The link to the survey was advertised through online local newspapers, and across the social media platforms of the Hamad Medical Corporation, which is the state funded major healthcare provider for the country. The advertisements were accompanied by short videos in English and Arabic explaining the rationale and nature for the survey. The survey was available in both English and Arabic languages.

### 2.2 Participants

All 2.3 million adult residents of Qatar^25^ were eligible for the study and were invited to participate in the survey.

### 2.3 Study materials

A validated vaccine hesitancy measurement tool—The Vaccine Attitudes Examination Scale (VAX) ^26^—was used as part of a composite questionnaire to assess the vaccine attitudes, awareness, and hesitancy among the study participants. This tool was translated into Arabic, and validation of the translated version was carried out using the guideline published by Sousa et al.^27^ The survey also collected relevant demographic and contextual information of the participants.

Details of study materials are given in the Appendix.

### 2.4 Outcome measures

The selection of study tools (VAX) and the design of the composite questionnaire were guided by the SAGE group recommendations in assessing vaccine hesitancy. These included:

*Contextual factors* like ethnic origin, gender, socioeconomic status, educational level, impact of media, individual's perception of the pharmaceutical industry among others.

*Individual and group influences* like previous vaccination experience, beliefs and attitudes to vaccination in general and knowledge and awareness of the COVID‐19 pandemic and vaccines, trust in health systems, and perception of risk and benefits of vaccines.

*Vaccine specific issues* like risks of new vaccine, risk to children and older adults, and role of healthcare professionals.

### 2.5 Ethical approval

The study was granted ethical approval by the Medical Research Council of the Hamad Medical Corporation. (MRC approval—01‐20‐930).

### 2.6 Analysis

We analyzed the data using descriptive statistics and multivariable logistic regression using Stata 12.^28^

## 3 RESULTS

We received 7859 responses to the survey; of these, 38 were under the age of 18 and were excluded from the analysis, leaving 7821 adult respondents. 66% of the respondents were in the working age group being between 26 and 45 years in age. 59.4% of the respondents were male, and 82.5% were married. 19.8% were healthcare workers; the remainder were from the general public.

11.7% were Qatari Nationals, and the remainder were Arab non‐Qatari (40.7%), Asian (38.1%), African (11.1%), European (7.1%), and American (North, Central and South) (3.1%). Over 76% were university educated. These figures are comparable to the general population composition in Qatar.

The median household size was 4 (IQR 2‐5). Over 77% were salaried, 11% were unemployed, 5.6% were self‐employed, and the remaining 6.2% were retired. 22.2% reported having a chronic physical illness, and 2.4% reported having a mental illness.

87.5% of respondents had completed childhood vaccinations, and 46.6% had received the influenza vaccine at least once in the last three years. 3.6% of respondents reported that they had had COVID‐19, and another 9.8% reported that a family member had had COVID‐19. (see Table 1).

**TABLE 1:** Demographic data and characteristics of participants (n = 7821)

	Frequency (%)
Respondent type	
Healthcare workers	1546 (19.77)
General public	6275 (80.23)
Age group	
18 to 25	261 (3.34)
26 to 35	2494 (31.89)
36 to 45	2666 (34.09)
46 to 55	1170 (14.96)
56 to 65	905 (11.57)
Over 65	325 (4.16)
Nationality	
Qatari	914 (11.69)
Non‐Qatari	6907 (88.31)
Educational level	
High school	813 (10.40)
University	6009 (76.83)
Trade/vocational/other	999 (12.77)
Occupation	
Salaried	6043 (77.27)
Self‐employed	436 (5.57)
Unemployed	859 (10.98)
Retired	483 (6.18)
Marital status	
Single	1362 (17.41)
Married	6459 (82.59)
Gender	
Male	4648 (59.43)
Female	3173 (40.57)
Childhood vaccination status	
Completed	6842 (87.48)
Not completed	446 (5.96)
No response	513 (6.56)
Received the seasonal flu vaccine in the last 3 y	
Yes	3646 (46.62)
No	3662 (46.82)
No response	513 (6.56)
Diagnosed with a chronic medical illness	
Yes	1740 (22.25)
No	5568 (71.19)
No response	513 (6.56)
Diagnosed with a mental illness	
Yes	191 (2.44)
No	7070 (90.40)
No response	560 (7.16)
Taking a regular medication	
Yes	2368 (30.28)
No	4625 (59.14)
No response	828 (10.59)
You or family member had COVID‐19 infection	
Yes	937 (11.98)
No	6056 (77.43)
No response	828 (10.59)

### 3.1 Intention to accept vaccine

In response to the question “Will you take the COVID‐19 vaccine when it becomes available?” 44.7% and 15.8% responded that they would “definitely” or “probably” accept the vaccine, respectively. 19.8% were unsure, 8.7% responded that they would “probably not” accept the vaccine, and 11.5% reported they would “definitely not” take the vaccine.

Very similar proportions were observed in response to a question on whether they would recommend the vaccine to elderly family members or relatives with chronic conditions, or whether they would get their children vaccinated for COVID‐19 (see Table 2).

**TABLE 2:** Intention to accept COVID‐19 vaccine

	Frequency	(%)
Will you have the vaccine when it becomes available		
Definitely	3123	(44.66)
Probably	1106	(15.82)
Not sure	1384	(19.79)
Probably not	606	(8.67)
Definitely not	774	(11.07)
Will you recommend vaccine to elderly family members?		
Definitely	3208	(46.76)
Probably	1175	(17.13)
Not sure	1418	(20.67)
Probably not	473	(6.89)
Definitely not	587	(8.56)
Will you get your children vaccinated for COVID‐19		
Definitely	2874	(41.89)
Probably	1090	(15.89)
Not sure	1419	(20.68)
Probably not	628	(9.15)
Definitely not	850	(12.39)

For those wishing to travel, 25.6% responded that they would preferentially accept the State required (at that time) 2‐week quarantine on return, rather than accept the vaccine.

### 3.2 Main worries and attitudes around COVID‐19 infection and its vaccine

The biggest worries reported were of family members getting infected (53%) or personally getting infected (37.6%) (see Table 3).

**TABLE 3:** Worries around COVID‐19 infection and its vaccine

	Frequency	(%)
Worries about getting infected	2942	(37.62)
Worries about a family member getting infected	4127	(52.77)
Financial worries	1523	(19.47)
Job‐related worries	1821	(23.28)
Worries of unavailability of a vaccine	2621	(33.51)

Beliefs toward COVID‐19 infection, vaccination and immunity were further explored through a 5‐point Likert scale (see Table 4). 53.8% of the respondents expressed concerns about vaccine safety because of COVID‐19 being a new disease. A similar proportion of 47.9% expressed concerns about longer‐term safety of vaccines in general. 92.1% of the respondents expressed the belief that natural exposure to infections gave the safest protection.

**TABLE 4:** Beliefs toward COVID‐19 vaccine and immunity

	Frequency	(%)
COVID‐19 is not a real disease		
Strongly disagree	3499	(44.74)
Disagree	592	(7.57)
Neutral	846	(10.82)
Agree	433	(5.54)
Strongly agree	816	(10.43)
COVID is a new disease, and vaccines have not been fully tested		
Strongly disagree	833	(10.65)
Disagree	874	(11.18)
Neutral	1617	(20.68)
Agree	928	(11.87)
Strongly agree	1934	(24.73)
I feel safe after being vaccinated		
Strongly disagree	1140	(14.58)
Disagree	734	(9.38)
Neutral	1601	(20.47)
Agree	1223	(15.64)
Strongly agree	1488	(19.03)
I can rely on vaccines to stop serious diseases		
Strongly disagree	700	(8.95)
Disagree	648	(8.92)
Neutral	1404	(17.95)
Agree	1401	(17.91)
Strongly agree	2033	(25.99)
I feel protected after getting vaccinated		
Strongly disagree	788	(10.08)
Disagree	713	(9.12)
Neutral	1512	(19.33)
Agree	1483	(18.96)
Strongly agree	1690	(21.61)
Although most vaccines are safe, there may be problems		
Strongly disagree	279	(3.57)
Disagree	454	(5.80)
Neutral	1263	(16.15)
Agree	1531	(19.58)
Strongly agree	2659	(34.00)
Vaccines cause serious problems in children		
Strongly disagree	608	(7.77)
Disagree	768	(9.82)
Neutral	1846	(23.60)
Agree	1237	(15.82)
Strongly agree	1727	(22.08)
I worry about serious unknown effects of the vaccine in the future		
Strongly disagree	458	(5.86)
Disagree	587	(7.51)
Neutral	1353	(17.30)
Agree	1241	(15.30)
Strongly agree	2547	(32.57)
Vaccines make a lot of money for pharmaceutical companies		
Strongly disagree	1081	(13.82)
Disagree	944	(12.07)
Neutral	1549	(19.81)
Agree	918	(11.74)
Strongly agree	1694	(21.66)
Authorities promote vaccines for financial gain not for people's health		
Strongly disagree	2597	(33.21)
Disagree	1101	(14.08)
Neutral	1262	(16.14)
Agree	481	(6.15)
Strongly agree	745	(9.53)
Vaccination programs are a big con		
Strongly disagree	2396	(30.64)
Disagree	1064	(13.60)
Neutral	1516	(19.38)
Agree	470	(6.01)
Strongly agree	740	(9.46)
Natural immunity lasts longer than vaccination		
Strongly disagree	820	(10.48)
Disagree	742	(9.49)
Neutral	1674	(21.40)
Agree	1038	(13.27)
Strongly agree	1912	(24.45)
Natural exposure to germs and viruses gives the safest protection		
Strongly disagree	1312	(12.61)
Disagree	986	(23.90)
Neutral	1869	(12.79)
Agree	1000	(13.03)
Strongly agree	1019	(79.09)
Being exposed to diseases naturally is safer for the immune system than vaccination		
Strongly disagree	1355	(17.33)
Disagree	977	(12.49)
Neutral	1821	(23.28)
Agree	913	(11.67)
Strongly agree	1120	(14.32)

We categorized those who reported were “definitely” or “probably” not going accept a COVID‐19 vaccine as vaccine hesitators, and investigated variables associated with vaccine hesitancy. We first investigated univariate associations, and then carried out multivariable logistic regression and included all variables that were significant at *P* < .2 level of significance. We found that those who were significantly more likely to be vaccine hesitators were older, native Qataris, self‐employed or retired, single, and female (Table 5). Non‐locals of working age were significantly more likely to accept the vaccine in contrast with the nationals in the same age group. In fact, the overall vaccine hesitancy among the local Qataris of working age was 42.57% compared to 16.71% for the immigrant population (Table 6). Those who had a flu vaccine in the last 3 years, took regular medication were significantly less likely to be vaccine hesitators (see Table 5).

**TABLE 5:** Multivariate logistic regression model of variables associated with vaccination hesitancy

Variable	Odds ratio	95% Confidence Interval	*Z* value	*P* value
Health Worker	1	1.03‐1.72	2.24	.025
General Public	1.33			
Age	1.27	1.15‐1.40	4.98	<.001
Foreign Nationals	1			
Qatari Nationals	1.68	1.30‐2.16	4.03	<.001
High school educated	1			
University Educated	0.81	0.55‐1.19	−1.07	.283
Trade/vocational/other	1.11	0.83‐1.51	0.72	.473
Salaried	1			
Self‐employed	1.60	1.12‐2.29	2.56	.011
Unemployed	0.99	0.74‐1.31	−0.08	.933
Retired	1.08	0.73‐1.60	0.40	.687
Married	0.74	0.59‐ 0.92	−2.64	.008
Female gender	1.82	1.51‐2.20	6.28	<.001
Completed childhood vaccinations	1.21	0.84‐1.74	1.03	.304
Chronic physical illness	0.82	0.63‐1.07	−1.44	.149
Mental illness	1.21	0.72‐0.2.05	0.72	.472
Had the flu vaccine in the last 3 y	0.54	0.44‐0.64	−6.38	<.001
Taking regular medications	0.73	0.56‐0.93	−2.51	.012
Belief that COVID‐19 is not a real disease	1.08	1.02‐1.15	2.60	.009
Worries that COVID‐19 is a new disease and vaccines have not been fully tested	1.71	1.58‐1.85	12.93	<.001
Feel safe after being vaccinated	0.47	0.43‐0.51	−16.71	<.001
Believe can rely on vaccines to stop serious infectious diseases	0.97	0.89‐1.06	−0.60	.546
Feel protected after getting vaccinated	0.80	0.72‐0.88	−4.40	<.001
Belief that most vaccines are safe but there may be as yet undiscovered problems	1.06	0.96‐1.12	1.20	.232
Worries about unforeseen problems in children	1.10	0.99‐1.22	1.75	.079
Worries about unknown future effects	1.18	1.05‐1.31	2.78	.005
Belief that vaccines make a lot of money for pharma but not much for regular people	0.98	0.90‐1.07	−0.44	.659
Belief that authorities promote vaccines for financial gain	1.11	1.02‐1.20	2.56	.010
Belief that vaccination programs are a big con	1.03	0.96‐1.12	0.88	.380
Belief that natural immunity lasts longer than vaccinations	0.92	0.85‐1.01	−1.76	.078
Belief that natural exposure to germs and viruses give the safest protection	1.20	1.10‐1.32	4.00	<.001
Belief that being exposed to diseases naturally is safer for the immune system than vaccination	1.05	0.96‐1.16	1.08	.280

**TABLE 6:** Intention to accept vaccine among working age migrants vs working age natives (18‐65 years old)

Will you refuse the COVID‐19 vaccine?	Yes	No
Natives	335 (42.57%)	452 (57.43%)
Immigrants	990 (16.71%)	4935 (83.29%)

We then carried out logistic regression to model opinions associated with vaccine hesitancy and controlled for the above variables. We found that vaccine hesitancy was significantly associated with the belief that there has been insufficient testing of COVID‐19 vaccines (OR = 1.7, *P* < .001), the view that authorities are motivated by financial gain rather than health of people (OR 1.14, *P* = .03) and that natural exposure to germs and viruses gives the safest protection (OR 1.22, *P* < .01) (see Table 5).

Finally, we investigated variables that would give respondents more confidence in accepting the COVID‐19 vaccine (Table 7). Of those who were unsure and showed vaccine hesitancy, 36.1% and 43.4% respectively reported that their own understanding of the disease and vaccine was the main reason that would make them more confident to accept the vaccine. The figures for healthcare workers for the same variables were 37.7% and 49.7%, respectively. The “other” category contained free‐text responses, of which 172 (2.2%) of the respondents stated that they would not take it under any circumstance.

**TABLE 7:** Variables that would give more confidence in accepting the vaccine among hesitators and non‐hesitators

Variables	Vaccine acceptance N (%)			
	Yes	Unsure	No	Total
Endorsement by a doctor	1168 (31.3)	232 (19.5)	116 (9.2)	1516 (24.5)
Endorsement by a public figure	36 (0.97)	8 (0.7)	1 (0.1)	45 (0.7)
Endorsement by Ministry of Health	891 (23.8)	155 (13.0)	47 (3.7)	1093 (17.7)
Endorsement by WHO	795 (21.3)	153 (12.9)	53 (4.2)	1001 (16.2)
Recommendation by friends/family	98 (2.6)	115 (9.7)	85 (6.8)	298 (4.8)
Research	658 (17.6)	430 (36.1)	545 (43.4)	1633(26.4)
Other	92 (2.5)	98 (8.2)	410 (32.6)	600 (9.7)
Total	3738 (100)	1191 (100)	1257 (100)	6186 (100)

## 4 DISCUSSION

This is one of the largest population‐based studies, to date, that addresses attitudes toward vaccination in the context of the COVID‐19 pandemic. Additionally, this is the first study in the Middle East and Northern African (MENA) region looking at the attitudes to the prospective COVID‐19 vaccine in a majority economic immigrant population using validated instruments that measure vaccine hesitancy within the SAGE framework. The main finding of this study is that as many as 20% of those surveyed showed hesitancy toward getting vaccinated with a COVID‐19 vaccine and a further 20% were unsure whether they would accept the vaccination or not.

To put this in perspective, emerging evidence from the few studies that have investigated COVID‐19 vaccine hesitancy needs to be considered. A survey by the COCONEL group^20^ in March 2020, in France, found vaccine hesitancy rates of 26%. Fisher et al found vaccine hesitancy at 10.8% and 31.6% were unsure in a survey in the USA in April 2020.^17^ A Canadian study^19^ found hesitancy rates of 20%‐25% in May 2020, and one from Britain^18^ revealed hesitancy rates 9% with 27% being unsure. A global survey across 19 countries found the vaccine hesitancy rates at 28%, although the degree varied by country.^21^ In a survey in October 2020 across 15 countries by Ipsos, a market survey organization, it was noted that the rates of average hesitancy were around 27% and was actually increasing over the course of the pandemic; again, there was a variation the rates across the countries and regions.^22^ From the emerging evidence, it appears that the rates of hesitancy are relatively higher in high‐income countries compared to medium income countries. The medium‐income countries also show more trust in their governments which may be associated with higher acceptance rates.^21^, ^22^ Qatar is a high‐income country, but the majority of its population, 90%, are economic migrants from low‐ to medium‐income countries.^25^ This study found that the local Qatari population have significantly higher hesitancy rates comparable to high‐income countries whereas the economic migrants have significantly lower hesitancy rates giving an overall hesitancy rate which is somewhat lower than the global aggregate rates. It is possible that the migrant group sees vaccination as a means to return to full economic activity. Additionally, most of these economic migrants are young working age men who do not have families living with them in Qatar and do not have to worry about vaccine safety for their families leading to significantly lower vaccine hesitancy rates. It corresponds to or finding that the single biggest worry expressed by the respondents was worry of their family members getting infected.

Across the surveyed populations in the published studies above, higher degrees of hesitancy were associated with lower socioeconomic and educational attainment.^17^, ^19^, ^21^, ^22^ Female gender and ethnic minority status was associated with more hesitancy in high‐income countries only.^17^, ^19^, ^20^ This study found that higher vaccine hesitancy was associated with female gender, being a native and being over 65 years of age. These findings are in keeping with high‐income countries and probably reflect the behavior and attitudes of the high‐income natives of Qatar. This is likely to be because of the unique composition of the Qatari population where the females and above working age individuals largely belong to either native population or economic migrants of high socioeconomic status. Economic migrants of poor socioeconomic status are generally young men who do not have families living with them. Further, this study's findings that females and older adults are more likely to show vaccine hesitancy is worrying as females play a central role in children's vaccination^29^ and older people are at higher risk of severe complications from the infection.

In the published studies described above, a more or less consistent theme of concerns around the safety of the vaccine itself is emerging as the most prominent. This concern cuts across demographic variables and countries. These concerns range from possible unexplored side effects of the vaccine, beliefs about the disease itself, public perception of vaccine trials being rushed through, pharmaceutical companies profiteering from the vaccine and preferred reliance on natural immunity.^17^, ^18^, ^21^, ^22^ These findings were mirrored in our survey and concerns around vaccine safety and longer‐term side effects were significantly associated with vaccine hesitancy.

The theme of concerns around vaccine safety was also significantly associated with vaccine hesitancy in healthcare workers themselves in this study population. Although concerning, it has been noted previously that while healthcare providers should be the ones that instill confidence in immunization programs, a paradoxical increase in vaccine hesitancy has been noticed in healthcare workers themselves for vaccination programs in the past.^30^

These concerns need to be taken together with our finding that nearly two thirds of those surveyed in our study reported that they trusted their own research to arrive at decision‐making for accepting the vaccine in preference to endorsement by healthcare professionals. This attribute of relying on personal research cut across demographic, socioeconomic, and educational variables in our study. It is well established that the availability of instant online information has allowed more and more people to seek information by themselves and this has been no different to COVID‐19.^31^, ^32^ Not surprisingly there is an increasing focus on the role of the media and particularly social media in shaping public opinion around the COVID‐19 disease and the vaccine. Studies have highlighted how media platforms of particular political leanings shape public opinion significantly different from those of the opposing political persuasion.^33^ Social media with its instant communication and access to wide audiences when coupled with ability to express anonymously provides an immense potential for propagation of unverified and unvetted information. Further, algorithms within social media platforms allow users to follow content that agrees with their views and reject contrasting views leading to development of distinct communities that subscribe to specific opinions and ideologies.^34^ This has been shown to be associated with increasing negative attitudes to the COVID‐19 and the vaccination.^32^, ^34^, ^35^

Our research further underlines the importance of developing trust in the safety of this vaccine. States and healthcare authorities need to recognize the power and influence of social media and devise innovative awareness and information dissemination strategies to increase vaccine uptake. Our study identifies the specific subgroups that these campaigns should be focused upon and the content of such campaigns.

Finally, there are limited previous data on vaccine hesitancy in Qatar. While there have been no population‐based studies, two studies have explored the uptake of and attitudes to seasonal Flu vaccination among healthcare workers. These studies report low levels of vaccine acceptance among healthcare workers with the most prominent concerns being the safety and side effects of vaccination.^36^, ^37^ While the acceptance rates for COVID‐19 vaccine are higher in this study compared to these two, it is interesting to note that as many as 12.7% of respondents in this study, who would usually take their Flu vaccine, were vaccine hesitant indicating that different factors may be at play here which need to be further explored.

Overall, the high degrees of hesitancy demonstrated should be of major public health concern when considering the minimum required proportion of the population needed vaccinate in order to achieve herd immunity. The frontrunners among the COVID‐19 vaccines in production are reporting efficiencies ranging between 70% and 80%, and with the reported R_0_ from pooled studies for COVID‐19 being 2.5 to 3.5, the percentage of the population that needs to be vaccinated to achieve herd immunity is between 70% and 90%^38^ underlining the magnitude of the task.

Moreover, it is known that vaccine uptake may be actually lower than the stated intent^39^ so the expected impact of vaccine hesitancy on an immunization program may be far worse than anticipated. Therefore, possible determinants of vaccine hesitancy in this region require in‐depth exploration.

### 4.1 Strengths

We surveyed a large nationally representative sample which allows a degree of generalizability of the results. Moreover, this study was conducted in a demographically distinct part of the world with a migrant‐majority population. Our study was conducted at the time when the front runners for COVID‐19 vaccine were publishing efficiency results and states across the world were discussing mass immunization strategies. A validated vaccine hesitancy tool was used, and outcome measures were based on internationally established vaccine hesitancy parameters.

### 4.2 Limitations

Our sample was somewhat self‐selecting as the study was available only in two languages which although widely spoken throughout the state of Qatar still excludes some non‐speaking residents. Additionally, Internet access was required to participate. This survey was conducted before the actual vaccination programs were rolled out and the hesitancy rates and attitudes are likely to vary as the situation evolves.

## 5 CONCLUSION

Vaccine hesitancy has obvious repercussions for the success of planned immunization initiatives and has been recognized as a threat to universal immunization programs and across the globe.^13^ In fact, WHO estimates that around 1 in 5 children do not receive routine lifesaving immunizations and as a result an estimated 1.5 million children still die each year of diseases that could be prevented by vaccines that already exist due to vaccine hesitancy.^13^

We found that a significant proportion of the respondents in Qatar showed vaccine hesitancy to the COVID‐19 vaccine, and although the vaccine hesitancy was lower in economic immigrants, the hesitancy attitudes were almost always driven by concern around the vaccine safety. The reliance on personal research to seek information underlines the role of social media in playing a significant part in influencing people's attitudes toward vaccine uptake. States and healthcare authorities need to recognize the massive trust deficit around the vaccine and use the popular media used by people to share credible and reliable information.

## CONFLICT OF INTEREST

None.

## AUTHOR CONTRIBUTIONS

Majid Alabdulla and Shuja Reagu are joint first authors of this work.

**Majid Alabdulla:** Conceptualization (equal); Investigation (equal); Methodology (equal); Project administration (equal); Resources (equal); Writing‐review & editing (equal). **Shuja Reagu:** Conceptualization (equal); Data curation (supporting); Formal analysis (equal); Funding acquisition (equal); Investigation (equal); Methodology (equal); Project administration (equal); Resources (supporting); Validation (equal); Writing‐original draft (lead); Writing‐review & editing (equal). **Abdullatif Al Khal:** Conceptualization (supporting); Investigation (supporting); Methodology (supporting); Project administration (supporting); Resources (equal); Writing‐review & editing (supporting). **Marwa Elzain:** Data curation (equal); Methodology (supporting); Project administration (equal); Resources (supporting); Writing‐review & editing (supporting). **Roland Jones:** Formal analysis (lead); Investigation (supporting); Software (lead); Validation (equal); Writing‐review & editing (equal).

## PATIENT AND PUBLIC INVOLVEMENT STATEMENT

There was no Patient or Public involvement in the design or recruitment of this study.

### PEER REVIEW

The peer review history for this article is available at https://publons.com/publon/10.1111/irv.12847.

## Supporting information

Supplementary Material

## Data Availability

The data that support the findings of this study are available from the corresponding author upon reasonable request and pending additional ethical approval.
