# Supplementary material for: COVID‐19 vaccine hesitancy and attitudes in Qatar: A national cross‐sectional survey of a migrant‐majority population
Source: Influenza Other Respir Viruses. 2021 Feb 19;15(3):361–70. doi: 10.1111/irv.12847 (PMC8014858; doi:10.1111/irv.12847)
Supplement: Supplementary file 1 — Supplementary Material [file IRV-15-361-s002.docx]

للاستعمال الرسمي فقط

| الرقم | التاريخ | نوع المقابلة:  مقدم للخدمات الصحية  فرد من عموم المجتمع. |
| --- | --- | --- |
|  |  |  |
|  |  |  |

بداية الاستبيان:

| المهنة | المستوى التعليمي | الجنسية | الجنس/ النوع | العمر |
| --- | --- | --- | --- | --- |
|  |  |  |  |  |

|  | الحالة الاجتماعية |
| --- | --- |
|  | عدد أفراد العائلة/الأشخاص الذين يشاركونك نفس المنزل؟ |

|  | هل أنتِ حامل أو مرضعة؟ |
| --- | --- |
|  | إذا كنتِ حامل، كم مدة الحمل بالأسابيع؟ |

| - نعم - لا | هل أكملت لقاحات الطفولة؟ |
| --- | --- |
| - سنويا - مرتين - مرة - لم تتلقاه | كم مرة تلقيت لقاح الإنفلونزا السنوي في السنوات الثلاث الأخيرة؟ |

| - نعم - لا | هل تم تشخيصك بأي أمراض مزمنة؟ |
| --- | --- |
| - السكري - ارتفاع ضغط الدم - ارتفاع نسبة الدهون - الربو - أمراض القلب (نقص التروية) - أخرى: الرجاء ذكرها | في حال الإجابة بنعم، الرجاء ذكر التفاصيل (اختر كل الإجابات المناسبة) |
| - نعم - لا | هل تم تشخيصك بأي أمراض نفسية؟ |
| - الاكتئاب - القلق - اضطراب المزاج ثنائي القطب - اضطراب ذهاني/الشيزوفرانيا - أخرى: الرجاء ذكرها | في حال الإجابة بنعم، الرجاء ذكر التفاصيل (اختر كل الإجابات المناسبة) |

| - نعم - لا | هل تتناول أي أدوية بصفة مستمرة؟ |
| --- | --- |
|  | في حال الإجابة بنعم، الرجاء ذكرها: |
| - لم أصب أو أي أحد من أفراد عائلتي بكوفيد-19 - لقد أصبت بكوفيد-19- - أصيب أحد أفراد عائلتي بكوفيد-19 - أصبت أنا وشخص اخر على الأقل من عائلتي بكوفيد-19 | هل أصبت أنت أو أي فرد من عائلتك بكوفيد-19 |
| - الخوف من أن أصاب بالعدوى - الخوف من أن يصاب أحد أفراد عائلتي بالعدوى - مخاوف مالية - مخاوف متعلقة بوظيفتك - عدم توفر لقاح حتى الآن - أنا قلق بعض الشيء - أنا لست قلق مطلقاً - أخرى | ما أكثر ما يثير قلقك بخصوص وباء كوفيد-19 ؟ (اختر كل ما ينطبق عليك) |

| - نعم بالتأكيد (الرجاء الإجابة على أسئلة الرغبة بتلقي اللقاح أدناه) - - نعم على الأغلب (الرجاء الإجابة على أسئلة الرغبة بتلقي اللقاح أدناه) - - لست متأكدا - لا على الأغلب - بالتأكيد لا | هل ستتلقى لقاح كوفيد-19 في حال توفره؟ |
| --- | --- |
| - نعم بالتأكيد - نعم على الأغلب - لست متأكداً - لا على الأغلب - لا بالتأكيد | هل ستوصي أفراد العائلة الكبار في السن أو الذين يعانون من أمراض مزمنة بتلقي لقاح كوفيد-19؟ |
| - نعم بالتأكيد - نعم على الأغلب - لست متأكداً - لا على الأغلب - لا بالتأكيد | في حال كان لديك أطفال، هل ستحرص على تلقيهم لقاح كوفيد-19 في حال توفره |
| - سأتلقى اللقاح بالتأكيد - سأتلقى اللقاح على الأغلب - لن أتلقى اللقاح وأفضل أن أخضع لإجراءات الحجر الصحي | اذا كنت ترغب بالسفر وكان البلد الذي ترغب بالسفر إليه سيتنازل عن فترة الأسبوعين من الحجر الصحي للأفراد الذين تلقوا لقاح كوفيد-19 فهل ستتلقى اللقاح؟ |

| - معرفتي بالمرض واللقاح - المعلومات التي حصلت عليها من الطبيب أو المستشفى - المعلومات التي حصلت عليها من وسائل التواصل الاجتماعي - المعلومات التي حصلت عليها من نشرات الأخبار - المعلومات التي حصلت عليها من العائلة أو الأصدقاء | ما هو السبب الرئيسي لرغبتك بتلقي اللقاح؟ |
| --- | --- |

الأسئلة التالية مصممة لتساعدنا على فهم معتقدات الأفراد حول تلقي اللقاحات بصورة أفضل. الرجاء منك أن تضع علامة على الاختيار الذي يعبر بصورة أفضل عن معتقداتك ومشاعرك. لا توجد إجابات صحية أو خاطئة:

| أوافق بشدة لا أوافق بشدة  1 2 3 4 5 6 | كوفيد -19 ليس مرضاً حقيقياً. |
| --- | --- |
| أوافق بشدة لا أوافق بشدة  1 2 3 4 5 6 | لقاحات كوفيد -19 ليست فعالة. |
| أوافق بشدة لا أوافق بشدة  1 2 3 4 5 6 | كوفيد19 مرض جديد ولم يتم اجراء اختبارات كافية على اللقاحات الخاصة به ولن تكون آمنة. |
| أوافق بشدة لا أوافق بشدة  1 2 3 4 5 6 | أشعر بالأمان بعد تلقي اللقاحات |
| أوافق بشدة لا أوافق بشدة  1 2 3 4 5 6 | أستطيع أن أعتمد على اللقاحات في وقف انتشار الأمراض المعدية |
| أوافق بشدة لا أوافق بشدة  1 2 3 4 5 6 | أشعر بأني محمي بعد تلقي اللقاحات. |
| أوافق بشدة لا أوافق بشدة  1 2 3 4 5 6 | بالرغم من أن معظم اللقاحات تبدو آمنة إلا أنه قد توجد بعض المشاكل التي لم نكتشفها بعد |
| أوافق بشدة لا أوافق بشدة  1 2 3 4 5 6 | قد تسبب اللقاحات ببعض المشاكل غير المتوقعة لدى الأطفال. |
| أوافق بشدة لا أوافق بشدة  1 2 3 4 5 6 | أشعر بالقلق حول التأثيرات غير المعروفة للقاحات في المستقبل |
| أوافق بشدة لا أوافق بشدة  1 2 3 4 5 6 | لدى اللقاحات عوائد مالية كبيرة لشركات الأدوية لكنها لا تعود بنفع كبير على الناس العاديين |
| أوافق بشدة لا أوافق بشدة  1 2 3 4 5 6 | تروج السلطات للقاحات بغرض النفع المادي وليس بغرض الحفاظ على صحة الناس |
| أوافق بشدة لا أوافق بشدة  1 2 3 4 5 6 | برامج التطعيم هي خدعة كبيرة |
| أوافق بشدة لا أوافق بشدة  1 2 3 4 5 6 | تدوم المناعة الطبيعية أكثر من المناعة المكتسبة من اللقاحات. |
| أوافق بشدة لا أوافق بشدة  1 2 3 4 5 6 | التعرض الطبيعي للفيروسات والجراثيم يعطي أكثر حماية آمنة |
| أوافق بشدة لا أوافق بشدة  1 2 3 4 5 6 | التعرض للأمراض بصورة طبيعية أكثر أماناً للجهاز المناعي من التعرض لها من خلال للقاحات. |

| - توصية طبيبي أو مؤسسة حمد الطبية أو مؤسسة الرعاية الصحية الأولية. - توصية من شخصية عامة. - توصية من وزارة الصحة - توصية من منظمة الصحة العالمية - ردود فعل إيجابية من أصدقائي أو أفراد عائلتي. - الاطلاع على دراسات حول فاعليته. - أخرى، الرجاء التفصيل: | ما الذي قد يجعلك أكثر ثقة لتقبل تلقي اللقاح؟ |
| --- | --- |
