# Supplementary material for: COVID‐19 vaccine hesitancy and attitudes in Qatar: A national cross‐sectional survey of a migrant‐majority population
Source: Influenza Other Respir Viruses. 2021 Feb 19;15(3):361–70. doi: 10.1111/irv.12847 (PMC8014858; doi:10.1111/irv.12847)
Supplement: Supplementary file 2 [file IRV-15-361-s001.docx]

***For office use only***

| Type of encounter   - Healthcare worker - General public | Date | Number |
| --- | --- | --- |
|  |  |  |

**Start of Questionnaire**

| Age | Gender | Nationality | Educational level | Occupation |
| --- | --- | --- | --- | --- |
|  |  |  |  |  |

| Marital status |  |
| --- | --- |
| How many family members/ individuals share your house? |  |

| Are you pregnant or breastfeeding? |  |
| --- | --- |
| If pregnant, How many weeks? |  |

| Have you completed your childhood vaccinations? | Yes No |
| --- | --- |
| How often have you received the annual influenza vaccine in the past 3 years? | - Annually - Twice - Once - Never |

| Do you have any chronic medical illness? | Yes No |
| --- | --- |
| If yes, please specify (tick all that apply) | - Diabetes - Hypertension - Dyslipidemia - Asthma - Ischemic heart disease - Other: specify- |
| Do you have any mental health illness? | Yes No |
| If yes, please specify (tick all that apply) | - Depression - Anxiety - Bipolar Illness - Psychosis/Schizophrenia - Other: specify- |

| Are you on any regular medication? | Yes No |
| --- | --- |
| If yes, please specify |  |
| Have you or a family member had COVID-19? | - I have had COVID-19 - A family member has had COVID-19 - Myself and at least one family member have had COVID-19 - No, neither myself or a family member have had COVID-19 |
| What are you most worried about during this Covid-19 pandemic? Tick all that apply | - Fear of becoming infected myself - Fear of a family member becoming infected - Financial worries - Job related worries - No available vaccination yet - Somewhat worried - Not worried at all - Other |

| Will you take the Covid-19 Vaccine when it becomes available? | - Definitely (answer willingness Q below) - Probably (answer willingness Q below) - Not sure - Probably not - Definitely not |
| --- | --- |
| When it becomes available, will you recommend the COVID-19 vaccine to elderly family members or family members with chronic conditions? | - Definitely - Probably - Not sure - Probably not - Definitely not |
| If you have children, will you get your children vaccinated for Covid-19 when it becomes available? | - Definitely - Probably - Not sure - Probably not - Definitely not |
| If you want to travel and the country of destination will waive the 2 weeks quarantine period for those who got Covid-19 vaccine, would you take the vaccine | - I would definitely take the vaccine - I would probably take the vaccine - I would not take the vaccine and prefer to go through the quarantine requirements |

| What is the main reason for your willingness? | - My understanding of the disease and vaccination - Information from my doctor/hospital - Information from social media - Information from news - Information from family/friends |
| --- | --- |
| These questions are designed to help us better understand people’s beliefs about vaccinations. Please mark the choices that most accurately reflect your feelings or beliefs. There are no right or wrong responses. | |
| Covid-19 is **not** a real disease | Strongly Strongly Disagree Agree  1 2 3 4 5 6 |
| Covid-19 is a new disease and vaccines against it have not been fully tested and will not be safe | Strongly Strongly Disagree Agree  1 2 3 4 5 6 |
| \| I feel safe after being vaccinated \| \| --- \| | Strongly Strongly Disagree Agree  1 2 3 4 5 6 |
| \| I can rely on vaccines to stop serious infectious diseases \| \| --- \| | Strongly Strongly Disagree Agree  1 2 3 4 5 6 |
| \| I feel protected after getting vaccinated \| \| --- \| | Strongly Strongly Disagree Agree  1 2 3 4 5 6 |
| \| Although most vaccines appear to be safe, there may be problems that we haven’t yet discovered \| \| --- \| | Strongly Strongly Disagree Agree  1 2 3 4 5 6 |
| \| Vaccines can cause unforeseen problems in children \| \| --- \| | Strongly Strongly Disagree Agree  1 2 3 4 5 6 |
| I worry about the unknown effects of vaccines in the future | Strongly Strongly Disagree Agree  1 2 3 4 5 6 |
| \| Vaccines make a lot of money for pharmaceutical companies, but don’t do much for regular people. \| \| --- \| | Strongly Strongly Disagree Agree  1 2 3 4 5 6 |
| \| Authorities promote vaccination for financial gain, not for people’s health \| \| --- \| | Strongly Strongly Disagree Agree  1 2 3 4 5 6 |
| \| Vaccination programs are a big con \| \| --- \| | Strongly Strongly Disagree Agree  1 2 3 4 5 6 |
| \| Natural immunity lasts longer than a vaccination \| \| --- \| | Strongly Strongly Disagree Agree  1 2 3 4 5 6 |
| \| Natural exposure to viruses and germs gives the safest protection. \| \| --- \| | Strongly Strongly Disagree Agree  1 2 3 4 5 6 |
| \| Being exposed to diseases naturally is safer for the immune system than being exposed through vaccination. \| \| --- \| | Strongly Strongly Disagree Agree  1 2 3 4 5 6 |

| What would make you more confident in accepting the vaccine? | - Endorsement by my doctor/HMC/PHCC - Endorsement by a public figure - Endorsement by Ministry of Health - Endorsement by WHO - Positive feedback from friends/family - Reading scientific research of its effectiveness - Other, specify- |
| --- | --- |
